# Monitoring Survivability and Infectivity of Porcine Epidemic Diarrhea Virus (PEDv) in the Infected On-Farm Earthen Manure Storages (EMS)

**DOI:** 10.3389/fmicb.2016.00265

**Published:** 2016-03-09

**Authors:** Hein M. Tun, Zhangbin Cai, Ehsan Khafipour

**Affiliations:** ^1^Department of Animal Science, University of Manitoba, WinnipegMB, Canada; ^2^Department of Medical Microbiology, University of Manitoba, WinnipegMB, Canada

**Keywords:** Swine, porcine epidemic diarrhea virus (PEDv), earthen manure storages (EMS), survivability, infectivity

## Abstract

In recent years, porcine epidemic diarrhea virus (PEDv) has caused major epidemics, which has been a burden to North America’s swine industry. Low infectious dose and high viability in the environment are major challenges in eradication of this virus. To further understand the viability of PEDv in the infected manure, we longitudinally monitored survivability and infectivity of PEDv in two open earthen manure storages (EMS; previously referred to as lagoon) from two different infected swine farms identified in the province of Manitoba, Canada. Our study revealed that PEDv could survive up to 9 months in the infected EMS after the initial outbreak in the farm. The viral load varied among different layers of the EMS with an average of 1.1 × 10^5^ copies/ml of EMS, independent of EMS temperature and pH. In both studied EMS, the evidence of viral replication was observed through increased viral load in the later weeks of the samplings while there was no new influx of infected manure into the EMS, which was suggestive of presence of potential alternative hosts for PEDv within the EMS. Decreasing infectivity of virus over time irrespective of increased viral load suggested the possibility of PEDv evolution within the EMS and perhaps in the new host that negatively impacted virus infectivity. Viral load in the top layer of the EMS was low and mostly non-infective suggesting that environmental factors, such as UV and sunlight, could diminish the replicability and infectivity of the virus. Thus, frequent agitation of the EMS that could expose virus to UV and sunlight might be a potential strategy for reduction of PEDv load and infectivity in the infected EMS.

## Introduction

Porcine epidemic diarrhea virus (PEDv), a highly contagious virus that causes severe diarrhea, vomiting, dehydration and high mortality particularly in piglets, is an enveloped, single-stranded RNA virus under the Coronaviridae family (Pensaert and de Bouck, 1978; Debouck and Pensaert, 1980; Song and Park, 2012). In late 1970, PEDv was first identified in the UK and Belgium (Wood, 1977; Pensaert and de Bouck, 1978). It has been reported in other European countries and Asia over the past four decades (Song and Park, 2012). In 2013, PEDv was first identified in the US, which genetically was highly similar to the Chinese virulence prototype isolated in 2012 (Stevenson et al., 2013). Subsequently, the virus has spread rapidly within North America, and the first introduction into Canada was reported in early 2014 (Kochhar, 2014; Pasick et al., 2014; Ojkic et al., 2015). Fecal-oral route presents the major transmission route for PEDv, however, airborne dissemination has been proposed as a potential additional transmission route because the virus can be aerosolized and transported over long distances (up to 10 miles downwind) by air (Alonso et al., 2014). In addition, fecal contamination of PEDv can cause fomites, for instance farm equipment such as transport trailers (Lowe et al., 2014) or feed supplements (Pasick et al., 2014) that act as potential abiotic carriers for PEDv. Fecal shedding of PEDv in pigs appears prior to clinical presentations, and hence increases the risk of transmission (Madson et al., 2014).

Environmental factors especially temperature and pH influence the survivability of virus in contaminated environments (Pesaro et al., 1995; Pujols and Segales, 2014). PEDv is able to survive up to 3 weeks at 4°C, 2 weeks at 12°C, 1 week at 22°C in spray dried plasma (Pujols and Segales, 2014), which was epidemiologically recognized as the source of virus infection for the first introduction into Canada (Pasick et al., 2014). Due to significant fecal shedding and the highly persistent nature of PEDv, proper storage, treatment and utilization of infected manure are important to prevent further contamination of uninfected environments.

Earthen manure storages (EMS; previously referred to as lagoon) are engineered structures for storage and treatment of liquid livestock manure. Swine EMS are mostly anaerobic, allowing anaerobic bacteria to decompose organic materials (Cole et al., 2000). Anaerobic EMS can significantly reduce pathogen concentrations including bacteria, viruses and parasites (Hill and Sobsey, 2001). However, the persistence of viruses in EMS may be prolonged if no proper treatment is employed (Costantini et al., 2007). Dee et al. (2005) found that porcine reproductive and respiratory syndrome virus (PRRSv) could survive in the infected EMS at 4°C for up to 8 days. In addition to PEDv, swine hepatitis E virus (HEV) has been reported to survive in infected EMS (Kase et al., 2009). Viruses in EMS can be expected to contaminate the uninfected environment via soil, ground and surface water and bioaerosols, from which they may infect other susceptible hosts (Cole et al., 2000). In western Canada, open farm anaerobic EMS are commonly used to store and treat the manure in most swine farms. To date (January 2016) and in Manitoba, five cases of PEDv outbreaks have been reported since February 2014 with an evidence of recurrent infection in some farms. However, the exact length of PEDv survivability in infected EMS is still unknown. This is an important question to answer in order to allow farmers and manure applicators to take necessary precautions to avoid further disease spread between infected farms and farms without prior exposure.

In this study, we monitored the survivability and infectivity of PEDv in two infected on-farm EMS over a period of 9 months. Based on the survivability and infectivity results, we determined that PEDv is highly viable in the infected EMS beyond 6 months, but environmental factors, such as UV and sunlight, could perhaps diminish its infectivity. In the present study, the ability of PEDv to replicate in EMS provided a clue that the virus may find an alternative host(s) to replicate and evolve within the EMS. Further studies are warranted to confirm this finding.

## Materials and Methods

### Study Design

Two PEDv infected farms identified in the province of Manitoba in 2014 participated voluntarily in this study. An on-farm EMS in each infected farm was selected for sampling: the farm-1 EMS had an area of 78 m × 46 m with an average depth of 1.5 m whereas the farm-2 EMS had an area of 79 m × 73 m and average depth of 0.7 m. The PEDv outbreak at farm-1 was confirmed in May 2014 by Veterinary Diagnostic Services of Manitoba. Samples tested at the time included saliva, fecal swabs (live animal and manure) and environmental swabs from load out, entrance, and pits. All representative samples from these areas were positive. However, no testing was done on EMS samples. No other testing was done following these samplings and no other visits to the site occurred after May until sampling started for the current study in September of 2014. No more pigs entered the barn between May and the end of the project. At the time of site visit in September, pigs looked healthy, active without any signs of scouring.

The outbreak at farm-2 occurred in September 2014. Similarly, initial tests were done at Veterinary Diagnostic Services of Manitoba. Samples included saliva, fecal swabs (live animal and manure) and environmental swabs (load out, entrance). All representative samples from these areas were positive. However, all samples were again collected in-barn, and no EMS sampling/testing was performed.

Before barns were depopulated, the status of active viral shedding was examined in fresh fecal and pit samples collected from each barn. Samplings from fresh fecal and pits were started late September for farm-1 and early October 2014 for farm-2. Sampling was terminated if animals showed high viral shedding, however, continued for another two consecutive weeks if samples were negative or showed low positivity. Farm-1 was completely depopulated on September 28, 2014, and farm-2 was depopulated on October 8, 2014.

**FIGURE 1:**
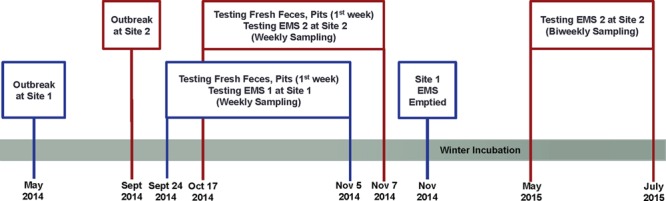
**Schematic diagram of the study design indicating porcine epidemic diarrhea virus (PEDv) outbreak and sampling schedule for monitoring survivability and infectivity of the virus in two on-farm Manitoba earthen manure storages (EMS)**.

To monitor the survivability and infectivity of PEDv, EMS samples were taken weekly before EMS were emptied. Details of the study design were illustrated in **Figure 1**.

### Biosecurity Measures

Strict biosecurity procedures were followed during planning, working on the site, transporting samples off-site, leaving the site, and during decommissioning to eliminate the risk of PEDv transmission. All protocols were established prior to accessing the sites to prevent further spread of the virus. A change tent was set up on the initial site visit to provide a location to add additional protective gear and site-specific footwear at a crossover point near the farm entrance. A research tent was also located to provide shelter during the sampling days and provide a location to store sampling and cleaning equipment. Specific route was identified to access the EMS, and separate vehicles were used for each farm to add an additional level of biosecurity. After sampling, samples were kept on dry ice and transferred to the vehicle. A specific biosecurity protocol for washing vehicles at the site as well as in the city was implemented as part of the sample hand off to the Gut Microbiome Laboratory (Department of Animal Science, University of Manitoba, Winnipeg, Manitoba, Canada) where further analyses were performed.

### Sampling Procedures, and Measurement of Temperature and pH of the EMS

**FIGURE 2:**
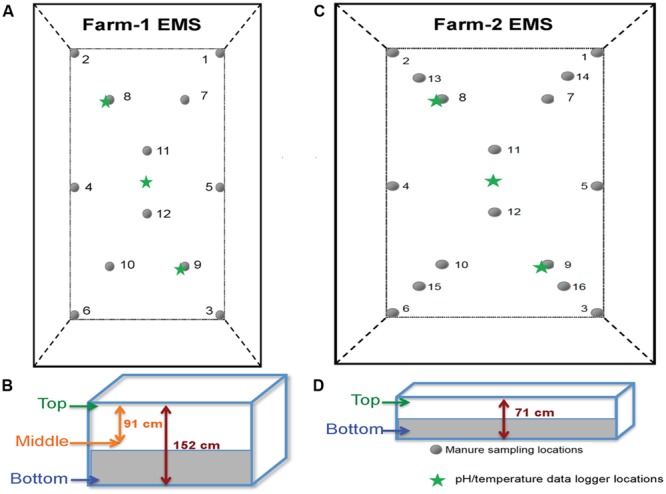
**The layout of sampled EMS in this study. (A)** The layout of farm-1 EMS indicating the locations of 12 sampling points and pH/temperature data loggers. **(B)** Three sampling depths at farm-1 EMS. **(C)** The layout of farm-2 EMS indicating the locations of 16 sampling points and pH/temperature data loggers. **(D)** Two sampling depths at farm-2 EMS.

Based on the EMS size, manure samples from farm-1 EMS were collected at 12 different locations and at three depths (*n* = 36/week). From farm-2 EMS, samples were collected at 16 different locations at two depths (*n* = 32/week; **Figures 2A,B**). In farm-1 EMS, data loggers were placed at three different locations, at three depths to continuously record the temperature and pH throughout the sampling period. However, in farm-2 EMS, data loggers were only placed at a single depth at three different locations due to shallow depth of the EMS (**Figures 2C,D**).

At the initial sampling, buoys tied to weights were used to mark the sampling locations in each EMS. Ropes were set up in a grid to mark the relative buoy location and allow researchers to maneuver the boat on the EMS with minimal agitation of the liquid. Samples were retrieved from the site in a double tote system to maintain biosecurity. A primary containment tote was taken to the change tent and left in the change tent while a cooler was taken to the EMS. The sample cooler and primary containment tote were placed in a secondary containment tote that stayed in the vehicle. Stainless steel samplers were custom fabricated for the EMS sampling, ranging in length from 2.1, 2.7, and 3.9 m. Samplers were made with a sample cup attached to shaft that slid through a pipe with sealing face. At the sampling location, the desired depth was measured prior to the sampling of each layer. Spring pressure held the sample cup closed until manually opened at the desired depth. Once opened, the manure flowed into the sample cup and then manually closed. The sample cup was retrieved and transferred the sample to a disposable sample jar for subsampling into vials. Manure samples were collected from two locations at a time before the samplers were brought back to shore for cleaning prior to the next set of samples being collected. The samplers were disassembled and the exteriors/interiors of all components were pressure washed prior to collecting the next set of samples. The manure samples were subdivided into vials and placed in the cooler with dry ice for transport back to the laboratory for testing. The gross appearances of EMS samples from different layers showed more liquid in the top layer while the bottom layer had more solid fraction.

### Nucleic Acid Preparation and Real-Time RT-PCR Assay

**FIGURE 3:**
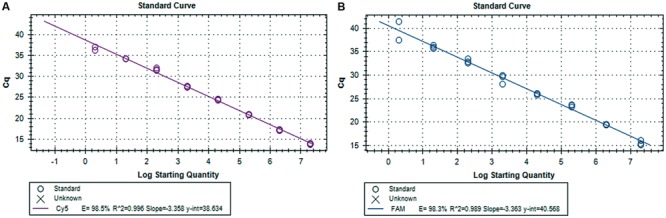
**Standard curves for the duplex real-time RT-PCR assay. (A)** Synthetic DNA standard curve for virulent PEDv strain. **(B)** Synthetic DNA standard curve for variant-INDEL PEDv strain.

The viral genomic RNA was extracted from 50 μl of sample using the MagMAX^TM^-96 Viral RNA Isolation Kit (Applied Biosystems, Foster City, CA, USA), according to the manufacturer’s instructions. RNA was then eluted in 90 μl of elution buffer. Viral RNA extracted from fecal, pit and EMS samples were subjected to molecular detection for both virulence (subgenogroup 2a) and variant-INDEL strains of PEDv using duplex real-time RT-PCR as described previously (Wang et al., 2014), which used spike gene primers (Forward S1F: AGG CGG TTC TTT TCA AAA TTT AAT G and Reverse S1R: GAA ATG CCA ATC TCA AAG CC) and specific probes targeted to virulent PEDv and new variant PEDv (Virulent S1P: 5Cy5-TAT TGG TGA AAA CCA GGG TGT CAA T-3BHQ-2, and Variant S1P: 56-FAM-TGG TTA TCT ACC TAG TAT GAA CTC CTC TAG C-3IABkFQ). The primer and hydrolysis probe utilized the AgPath-IDTM One-Step RT-PCR Reagents (Life Technologies, Grand Island, NY, USA) and 2 μL of RNA with the CFX348 Real-Time PCR System (Bio-Rad, Hercules, CA, USA) under the following thermal cycling conditions, reverse transcription, 30 min at 50°C; Taq activation, 15 min at 95°C; followed by 40 cycles of 10 s at 94°C and 30 s at 54°C. A 500 bp of spike gene fragment that included sequence variation between PEDv strain OH1414 (virulent PEDv) and PEDv strain OH851 (variant PEDv) was chosen to use as both standard and positive control. A gBlocks Gene Fragments (Integrated DNA Technologies, Inc., Coralville, IA, USA) containing the PEDv spike gene targets was synthesized. Stock concentration of 10^9^ copies/μl were made and a 10-fold serial dilution was run on the real-time RT-PCR to generate a standard curve for each genotype of PEDv, which was used to transform the *C*q values into estimated copies of PEDv RNA per ml of EMS. The sensitivity of the duplex real-time RT-PCR assay was validated through serial dilutions of both gene fragments, and triplicate of each dilution were run in the assay. The detection limit was two copies for both variant and virulent strains of PEDv. As shown in **Figure 3**, there is a strong linear correlation (*r*^2^ > 0.99) between *C*q values and the corresponding amount of gene fragment copy numbers for both virulent and variant PEDv. The standard curves of virulent and variant PEDv were plotted with slopes of -3.358 and -3.363, respectively. The amplification efficiencies of the assays for both virulent and variant PEDv were 98.5 and 98.3%, respectively (**Figures 3A,B**). Triplicate for each EMS sample were also subjected for duplex real-time RT-PCR assay, and generated *C*q values were transformed into copy numbers based on the slopes of respective standard curves. Subsequently, the resulted copy numbers were further transformed into copy numbers in 1ml of EMS sample.

### PEDv Infectivity in Cell Culture

To evaluate the infectivity of PEDv, EMS samples were centrifuged and filtrated using 0.2 micron syringe filter (VWR International Inc., Radnor, PA, USA; cat. no. 194-2520), then subjected to 10-fold serial dilution with phosphate buffer saline (PBS). The serially diluted samples were inoculated in VERO cells as previously described (Hofmann and Wyler, 1989; Chen et al., 2014). Briefly, VERO cells (ATCC CCL-81) in 96-well plate were washed twice with 100 μl PBS then inoculated with 200 μl of sample. Triplicate for each sample were tested in the presence of known positive control. After a 1.5 h incubation at 37°C with 5% CO2, 100 ml PBS was used to wash the cells once. Then, cells were incubated for 5 days with the post-inoculation medium. The post-inoculation medium was composed of MEM supplemented with tryptose phosphate broth (0.3%), yeast extract (0.02%), and trypsin 250 (5 μg/ml). The cytopathic effect (CPE) in the cell culture plates was monitored daily. After 5 days, the plate was frozen-and-thawed to detach cells from the plates. Cells were then subjected to viral RNA extraction to examin PEDv’s replicability using the duplex real-time RT-PCR assay (Wang et al., 2014). The virus titers were determined according to the Reed and Muench (1938) method and expressed as the 50% tissue culture infective dose (TCID50)/ml. Higher TCID50 values were indicative of higher infectivity of virus.

### Statistical Analyses

Data from the quantitative RT-PCR analysis, sampling date, different sample types and pathogen were consolidated in a spreadsheet (Microsoft EXCEL; Microsoft Corporation, Redmond, WA, USA) and organized for analysis. Means, standard deviations, and minimum and maximum values for quantitative variables, and positive samples counts and percentages for qualitative variables were calculated for descriptive analysis. Statistical analysis was performed on real-time RT-PCR results using chi-square, two-way ANOVA with Sidak’s multiple comparison test, and linear regression in Prism 5.0 (GraphPad Software Inc., San Diego, CA, USA). *P*-values < 0.05 were considered significant.

## Results

### The Status of PEDv Shedding in the Farms

To evaluate active viral shedding in the farms, we verified the presence of PEDv in both fresh fecal and pit samples using duplex qualitative RT-PCR assay. **Table 1** presents the status of virus shedding in fresh fecal and pit samples collected from barns before they were depopulated. In farm-1 (which contained two barns), minor PEDv shedding (only virulence PEDv strain) occurred in the barn A, 1 and 2 weeks before barn was depopulated (5 and 15%, respectively), whereas no viral shedding was detected in barn B. Pit samples in farm-1 showed higher PEDv positivity in barn A (100 and 90%) than in barn B (20 to 30%). In farm-2, pigs were actively shedding PEDv and 100% of tested pit and fecal samples were positive to virulence PEDv strain with average viral loads of 2.4 × 10^6^ copies/ml of manure mix in the pit and 3.2 × 10^6^ copies/g of feces, respectively. No variant-INDEL strain was detected in this study.

**Table 1:** Status of porcine epidemic diarrhea virus (PEDv) shedding before depopulation of barns by examining the presence of PEDv in fresh feces and pit samples.

Farm	Barn	Sample type	% of PEDv positive samples (Number of PEDv positive samples/total number of tested samples)			
			September 24th	October 1st	October 8th	October 17th
1	A	Fresh feces	15 (3/20)	5 (1/20)	Barn was emptied	
		Pit	100 (10/10)	90 (9/10)	Barn was emptied	
	B	Fresh feces	0 (0/10)	0 (0/10)	0 (0/10)	Barn was emptied
		Pit	20 (2/10)	30 (3/10)	20 (2/10)	Barn was emptied
2	NA^1^	Fresh feces	NA	NA	NA	100 (12/12)
		Pit	NA	NA	NA	100 (12/12)

*^1^NA, Not Applicable.*

### Dynamics of pH and Temperature in Earthen Manure Storages

**FIGURE 4:**
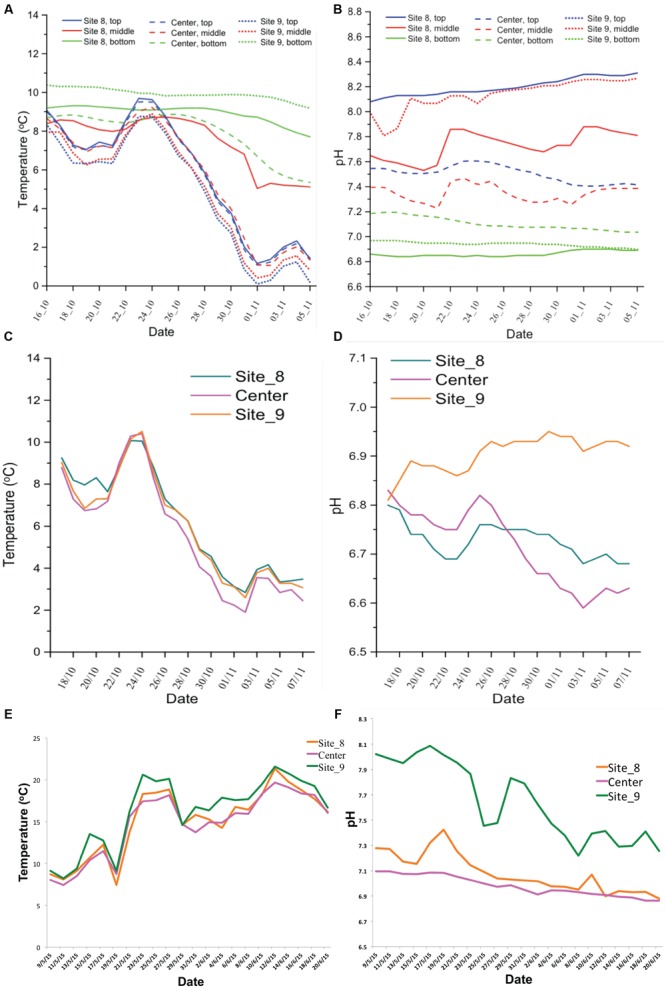
**Temperature and pH dynamics in the studied EMS.** The temperature and pH records for farm-1 EMS **(A,B)** and for farm-2 EMS **(C,D)** during fall sampling. The temperature and pH records for farm-2 EMS **(E,F)** during spring/summer sampling.

**Figure 4A** presents the temperature dynamics in farm-1 EMS during fall sampling. Data were retrieved from nine temperature/pH loggers that were set up at three different locations in three different depths were presented in **Figures 2A,B**. In all three layers of the EMS, the temperature was in the range 16–19°C at the beginning of the study in late September. However, the temperature of the top and middle layers of the EMS declined to 4–8°C by Oct 25th, followed by the second decline by mid-November to 0–2°C. The temperature of the bottom layer of the EMS remained at the range of 6–10.5°C until mid-November and was less impacted by the environmental temperature. **Figure 4B** presents the pH dynamics in farm-1 EMS where pH was relatively more stable in the bottom layer compared to the top and middle layers throughout the monitoring period. The bottom layer of the EMS also had the lowest pH ranging from 6.8 to 7.2. The top layer had the highest pH ranging from 7.5 to 8.4 while the middle layer had a pH of 7.2–8.3.

**Figures 4C,D** show the temperature and pH dynamics in farm-2 EMS during the fall and spring/summer sampling periods, respectively. Due to the shallow depth, data were retrieved from three loggers that were set up at three different locations of only at a single layer presented in **Figure 2D**. The temperature dynamic in farm-2 EMS followed a similar trend like in farm-1 EMS. The pH ranged from 6.6 to 6.95 which was similar to the pH observed in the bottom layer of farm-1 EMS. However, the temperature in farm-2 EMS steadily increased during spring/summer sampling (**Figure 4E**). Similarly, the pH showed an increase in spring and gradually decreased to 6.8–7.2 by the end of the sampling period (**Figure 4F**).

### Survivability of PEDv in the Infected on-Farm Earthen Manure Storages

**FIGURE 5:**
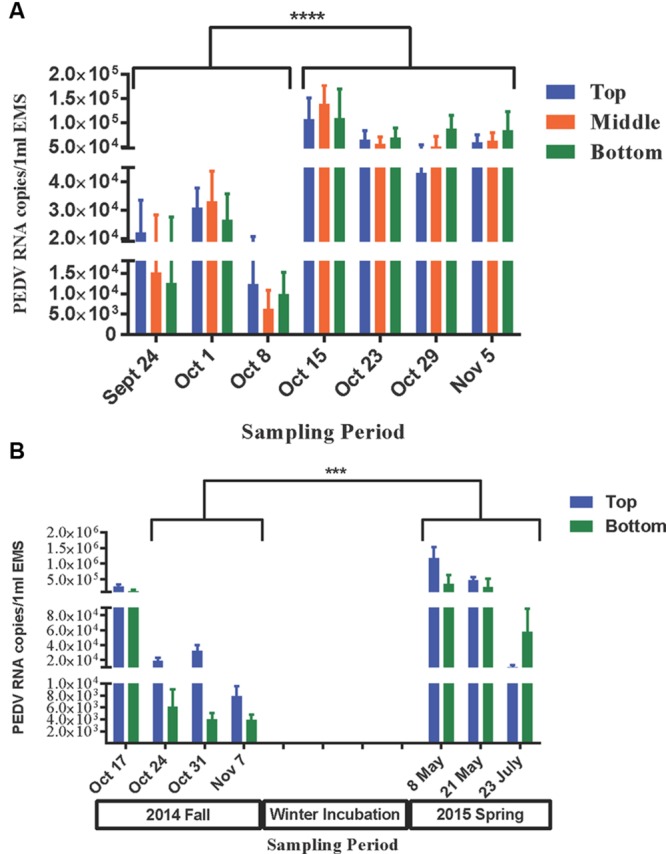
**Survivability of PEDv in two infected on-farm EMS.** Using real-time RT-PCR targeted to *S* gene, the survivability of PEDv over time was tested based on the detectable viral RNA copies number in 1 ml of EMS at each sampling time point during fall 2014 sampling for farm-1 EMS **(A)**, and from fall 2014 to summer 2015 sampling for farm-2 EMS **(B)**. The bar shows average RNA copy number of PEDv in the respective layer of EMS. The error bars show the standard deviation based on 12 replicates per layer of the EMS in farm-1 and 16 replicates in farm-2. Each biological sample was analyzed in triplicate using real-time RT-PCR. ^∗∗∗^*P* < 0.001 and ^∗∗∗∗^*P* < 0.0001.

The survivability of PEDv in the infected EMS was determined by the presence of viral RNA for both virulent PEDv (subgenogroup 2a) and variant-INDEL strains. In farm-1 EMS, 97% of tested EMS samples were PEDv positive only for virulent PEDv (subgenogroup 2a), whereas no sample was detected for the variant-INDEL strain. The viral load significantly increased after the third week of sampling in all three layers of the EMS. On average, the viral load ranged from 6.3 × 10^3^ to 3.3 × 10^4^ copies/ml of EMS during the first 3 weeks, however, that significantly increased (*P* < 0.05) to 4.3 × 10^4^–1.4 × 10^5^ during the last 4 weeks (**Figure 5A**).

In farm-2 EMS, 76% of tested EMS samples were positive for virulent PEDv (subgenogroup 2a), and no variant-INDEL strain was observed like in farm-1 EMS. During the fall sampling, viral load was higher (*P* < 0.05) at the first week of sampling (on average 1.26 × 10^5^–2.69 × 10^5^ copies/ml of EMS) compared to the following 3 weeks (on average 3.9 × 10^3^–7.8 × 10^3^ copies/ml of EMS on week 4; **Figure 5B**). After a long winter incubation, the viral load increased (*P* < 0.05) significantly in the early spring (May 2015) ranging, on average, from 3.53 × 10^5^ to 1.12 × 10^6^ copies/ml of EMS in the bottom and top layers of the EMS, respectively. However, the viral load significantly declined (*P* < 0.05) by mid-summer (July 2015) ranging, on average, from 1.02 × 10^4^ to 5.76 × 10^4^ copies/ml of EMS in the top and bottom layers, respectively (**Figure 5B**).

### Infectivity of PEDv in the Infected on-farm Earthen Manure Storages

To examine the infectivity of PEDv in the infected EMS, sets of samples from selected weeks were inoculated in VERO cell culture and their ability to replicate was examined. Samples from both weeks 5 and 7 of farm-1 EMS, and weeks 2 and 4 from fall sampling and weeks 1, 3, and 5 from spring/summer sampling of farm-2 EMS were selected for infectivity analysis (**Table 2**). In farm-1 EMS, PEDv was detectable in the top layer and its number increased during the last 4 weeks of sampling in the fall, however, the viruses were not infective (**Table 3**), whereas samples from both middle and bottom layers of the EMS were infective. In week 5, the percentage of infectivity in both middle and bottom layer were the same (8.3%), whereas the infectivity was higher in the bottom layer (10^8^ TCID_50_) compared to the middle layer (10^5^ TCID_50_) although no statistical differences were observed (*P* = 0.58). In week 7, a higher number of samples showed positive infectivity in the middle layer when compared to the bottom layer (*P* = 0.01; **Table 3**).

**Table 2:** Sampling information and analyses performed in this study.

Farm	Sampling weeks	Season	Sampling date	Performed tests
1	Week 1	Fall	September 24, 2014	Survivability
	Week 2	Fall	October 1, 2014	Survivability
	Week 3	Fall	October 8, 2014	Survivability
	Week 4	Fall	October 15, 2014	Survivability
	Week 5	Fall	October 23, 2014	Survivability and infectivity
	Week 6	Fall	October 29, 2014	Survivability
	Week 7	Fall	November 5, 2014	Survivability and infectivity

2	Week 1	Fall	October 17, 2014	Survivability
	Week 2	Fall	October 24, 2014	Survivability and nfectivity
	Week 3	Fall	October 31, 2014	Survivability
	Week 4	Fall	November 7, 2014	Survivability and infectivity
	Week 1	Spring	May 8, 2015	Survivability and infectivity
	Week 3	Spring	May 21, 2015	Survivability and infectivity
	Week 5	Summer	July 23, 2015	Survivability and infectivity

**Table 3:** Infectivity of PEDv in the farm-1 EMS.

Sampling week/date	EMS layer	% of PEDv infective (number of PEDv infective samples/ total number of tested samples)	Infective titer (TCID_50_)^1^	Fisher’s exact test^2^	
				*X*^2^	*P*-value
Week 5/23 October 2014	Top	0 (0/12)	0.0	1.06	0.58
	Middle	8.3 (1/12)	1.00E + 05		
	Bottom	8.3 (1/12)	1.00E + 08		
Week 7/05 November 2014	Top	0 (0/12)	0.0	8.4	0.01
	Middle	41 (5/12)	1.00E + 06 to 1.00E + 09		
	Bottom	8.3 (1/12)	1.00E + 08		

*^1^TCID_50_: the 50% tissue culture infective dose. Greater values are indicative of greater infectivity of virus.*

*^2^Statistical comparisons were made between different layers of the EMS within each sampling time point.*

In farm-2 EMS, the infective virus was found in both top and bottom layers during the fall sampling. However, only samples from the bottom layer of the EMS were found to be infective with a low titer of 10^2^ TCID_50_ during the spring samplings on May 8th and 21st. Both infectivity percentage and titer showed no significant difference between top and bottom layer of the EMS in the fall samples and the first week (May 8th) of spring sample. However, there was a significant difference in the second week (May 21st) of spring sample (*P* = 0.01; **Table 4**).

**Table 4:** Infectivity of PEDv in the farm-2 EMS.

Sampling week/date	EMS layer	% of PEDv infective (number of PEDv infective samples/ total number of tested samples)	Infective titer (TCID_50_)^1^	Fisher’s exact test^2^	
				*X*^2^	*P*-value
Week 2 fall/24 October 2014	Top	12.5 (2/16)	1.00E + 02 to 1.00E + 06	0	1
	Bottom	12.5 (2/16)	1.00E + 01 to 1.00E + 07		
Week 4 fall/07 November 2014	Top	6.25 (1/16)	1.00E + 02	1.14	0.28
	Bottom	18.75 (3/16)	1.00E + 02 to 1.00E + 04		
Week 1 spring/08 May 2015	Top	0 (0/16)	–	1.03	0.31
	Bottom	6.25 (1/16)	1.00E + 02		
Week 2 spring/21 May 2015	Top	0 (0/16)	–	6.36	0.01
	Bottom	31.25 (5/16)	1.00E + 02		
Week 3 summer/23 July 2015	Top	0 (0/16)	–	–	–
	Bottom	0 (0/16)	–		

*^1^TCID_50_: the 50% tissue culture infective dose. Greater values are indicative of greater infectivity of virus.*

*^2^Statistical comparisons were made between different layers of the EMS within each sampling time point.*

## Discussion

The primary route of infection for PEDv is oral-fecal transmission through direct contact with infected pigs or the infected manure (Crawford et al., 2015). Other potential routes of infection have been proposed (Alonso et al., 2014; Lowe et al., 2014; Pasick et al., 2014). Transport vehicles for swine, contaminated air and feed or feed ingredients have been shown to contain the genetic material of PEDv, which are indicative of highly persistence nature of PEDv outside the host. In general, most viruses cannot survive long in the environment, outside their host. However, viruses under the Coronaviridae family show high survivability in the contaminated environment (Geller et al., 2012). PEDv is an enveloped, single-stranded, positive-sense RNA virus belonging to the genus *Alphacoronavirinae* in the family Coronaviridae and is related to transmissible gastroenteritis virus (TGEV; Hofmann and Wyler, 1989). Persistence of viruses in the environment varies with the type of virus. Two coronaviruses including a swine pathogen, TGEV and mouse hepatitis virus (HMV) remain infectious in water and sewage from several days to weeks. At 25°C, TGEV survives up to 22 days and MHV survives up to 17 days in the water, whereas the survivability for TGEV is 9 and 7 days for MHV in settled sewage. At 4°C, both viruses can survive up to 4 weeks in water and sewage (Casanova et al., 2010). In case of canine coronavirus (CCV), it has lower survivability and loses its infectivity at 20°C and 4°C after 24 h, whereas complete loss of its infectivity is observed at -20°C or -70°C by 3 months (Tennant et al., 1994). Hepatitis A virus (HAV) is another example that is more stable than most enteroviruses at elevated temperatures (Deng and Cliver, 1995).

Experimentally, it was demonstrated that PEDv has high survivability in the infected manure (Goyal, 2013). However, so far there has been no epidemiological investigation of PEDv survivability in the infected manure over time in the environment. PEDv is a highly contagious virus with low infective dose (Liu et al., 2015). PEDv infected manure can contaminate the uninfected environment, making the manure storage methods and treatments critical to the control of this pathogen (Song and Park, 2012). In most swine farms, EMS are traditional manure storage and treatment system, and the manure is applied to the agricultural land as a valuable fertilizer source (Hunt et al., 2010; Ducey and Hunt, 2013). However, viruses shed in EMS are likely to contaminate the environment through soil, ground and surface water and bioaerosols, and therefore, application of infected manure could be a potential disease transmission source (Cole et al., 2000).

This study is the first field monitoring which examines the survivability of the PEDV in the infected EMS. In the lab-based observations, PEDv survives more than 7 days in the inoculated fresh feces, while the virus can survive up to 14 days at room temperature and up to 28 days at -20 to 4°C in inoculated manure slurry (Goyal, 2013). In another landmark study, PEDv has been experimentally reported to survive and be infectious up to 3 weeks at 4°C, 2 weeks at 12°C, and 1 week at 22°C in spray dried bovine plasma (Pujols and Segales, 2014). However, the experimental periods observed in both lab-based studies were limited and the observations were done only up to 28 and 21 days, respectively. Thus, the maximum duration PEDv can survive and be infectious in the environment was still unknown. Our current findings showed that PEDv could survive up to 9 months in the infected EMS (according to farm-2 EMS data) under fall winter, spring and summer temperatures in Manitoba (range of -30 to 23°C according to 2014–2015 weather records). The stability of coronaviruses at various temperatures appears to be dependent on the nature of surrounded environmental conditions. In general, coronaviruses can survive at 56°C for 10–15 mins, at 37°C for several days, and at 4°C for several months whilst virus at a frozen temperature (-60°C) survives for many years without loss of infectivity (Casanova et al., 2010). The survival time of most viruses in infected manure is highly variable but should be considered in terms of days, weeks, or months as opposed to minutes or hours. Enteroviruses are reported to survive for 3–170 days in the soil of various compositions at various temperatures and for 1–23 days on crops (Deng and Cliver, 1995). Survivability of viruses is substantially longer at cold temperatures. The average daily temperature of EMS would not be stable as in experimental setting, and is influenced by the ambient environmental temperature and other factors. Thus, PEDv survivability and infectivity in experimental settings cannot directly represent the exact environmental conditions. Based on our study, in particular, the results from farm-2 EMS, PEDv has the ability to survive and be infective up to 9 months after the outbreak in the farm. In addition, temperature fluctuations within a single day should also be considered to evaluate the impact on the survivability of PEDv in EMS.

Beside the temperature, pH of the environment has an impact on the survivability of virions. Generally, PEDv favors neutral pH with a wide range between 5 and 9 (Hofmann and Wyler, 1989). However the combined effect of pH and temperature plays a critical role in PEDv survivability. Although PEDv is active in the pH range between 5 and 9 at 4°C, the range narrows down with increased temperature to between 6 and 8 at 37°C. Regardless of temperature, the virion completely loses its replicability at pH < 4 and >pH 9 (Hofmann and Wyler, 1989). In this study, we observed that the variation of pH over time in both studied EMS were not significant, with the pH ranging between 6.6 and 8.3. Among three layers of EMS, pH was the lowest at the bottom layer and the highest in the top layer (**Figure 4**). The pH of both EMS in this study fell within the range that PEDv could actively replicate indicating that the EMS are favorable environment for PEDv replication. However, there was no significant direct correlation among pH, temperature, and viral copy numbers.

In both studied EMS, the viral load was numerically higher in the top layer during the first 3 weeks, however, the trend switched toward the end of the sampling period with the higher number observed in the bottom layer (**Figure 5**). This may be due to either progressively precipitation of virus in the EMS or differences in viral replication rates among different layers within an infected EMS over time.

In farm-1 EMS of this study, the viral load significantly increased after the third week of sampling in all three layers of the EMS. In the fall sampling of farm-2 EMS, viral load was higher at the first week of sampling compared to the following 3 weeks. This was most likely due to active viral shedding, which was confirmed by fresh fecal samples (**Table 1**) at the beginning of the study as the barn was depopulated about 2 weeks after EMS sampling was started. After a long-winter incubation, the viral load significantly increased in the early spring (May 2015), but significantly declined in mid summer (July 2015). Beyond the existing knowledge that PEDv significantly survives longer in contaminated environments, the apparent increased viral copies in both studied EMS provides evidence for the ability of the virus to replicate outside its typical host, the swine. Although PEDv is believed to be a genuine pig virus, its ability to replicate in cells of non-swine origin has been reported since late 1980. In 1988, Hofmann and Wyler (1989) were the first to report replication of PEDv in kidney cells of monkey origin (VERO cells). Subsequently, Utiger et al. (1995) presented serological evidence that PEDv may circulate in humans and cats. A recent report verified that PEDv could replicate in duck intestinal cell line (Khatri, 2015). To our knowledge, reservoirs of PEDv have not yet been discovered. Likewise, other swine viral pathogens could replicate in hosts of non-swine origin. However, in the current scenario, potential alternative hosts for PEDv in EMS are favored to be non-mammalian eukaryotes (e.g. protozoa or amoeba). EMS are natural inhabitants for most amoeba and protozoa in which a variety of viruses are able to replicate (Baron et al., 1980; Miles, 1988; Diamond, 1991; Wang and Wang, 1991a,b; Kasprzak and Majewska, 1995; Truong et al., 2013). Thus, further research is needed to investigate the ability of PEDv to replicate in non-mammalian eukaryotic hosts.

The presence of viral RNA in EMS simply indicated the presence of virion, while its replicability that reflects the infectivity, has been determined *in vitro* using cell culture bioassay in this study. To examine the replicability (infectivity) of PEDv in the studied EMS, samples were selectively monitored using VERO cell culture, which is a standard *in vitro* model for PEDv infectivity (Hofmann and Wyler, 1989; Khatri, 2015), however, the results obtained from such method may provide an underestimation compared to bioassays. In this study, the PEDv in farm-1 EMS showed no infectivity in the top layer, whereas certain infectivity was observed in the top layer of farm-2 EMS during fall, but not in spring/summer. These contradictory results could be explained by the differences in viral shedding status in two farms at the beginning of sampling. The virus shedding was active at the beginning of sampling in farm-2, which probably contributed to the infectivity of PEDv in the top layer of its EMS in the fall. Mostly, the top layer of both EMS had low or no infective PEDv, probably due to direct exposure of the environmental UV and sunlight that perhaps effectively reduced the infectivity of the virus. Generally, in both studied EMS, the infectivity titer of PEDv showed a gradual decrease in the later weeks of sampling. Combining survivability and infectivity data led us to hypothesize that although PEDv may have the ability to find an alternative host(s), and replicate in EMS, the virulence property of the virus might not stay the same when virus is replicating and evolving in a different host than swine. Therefore, further studies are needed to monitor the evolution of PEDv in infected EMS.

## Conclusion

In summary, this study furthers our existing knowledge of PEDv, indicating high survivability of this virus in the environment, especially in on-farm earthen manure storage system typical of western Canada. In practice, the non-infected farms may share manure applicators with other farms that have been infected with PEDv. Machines used for manure application are potential physical vectors (fomites), which can easily spread the virus if no proper disinfection is practiced. The handling and managing of infected manure is a critical component to reduce the risk of further virus transmission from one farm to others. Frequent agitations of EMS may allow direct exposure of infectious virions from the lower layers to environmental UV and sunlight, and reduce the infectious viruses in EMS, thus, probably decreasing the risk for recurrent infection within the farm and further spread of viruses. More research should be carried out to better understand the life cycle of PEDv in the environment, as well as its survivability outside the host. Disinfectants and treatment strategies for infected EMS should be reviewed in order to eradicate the PEDv from the environment. Additional studies are needed to monitor the survivability of PEDv in contaminated soils after application of infected manure onto agricultural land.

## Author Contributions

HT and EK conceived and designed the experiment. HT and ZC conduced the experiment and analyzed the data. All authors drafted the manuscript. All authors carefully read and approved the final version of the manuscript.

## Conflict of Interest Statement

The authors declare that the research was conducted in the absence of any commercial or financial relationships that could be construed as a potential conflict of interest.
